# Development of pVCR94ΔX from *Vibrio cholerae*, a prototype for studying multidrug resistant IncA/C conjugative plasmids

**DOI:** 10.3389/fmicb.2014.00044

**Published:** 2014-02-06

**Authors:** Nicolas Carraro, Maxime Sauvé, Dominick Matteau, Guillaume Lauzon, Sébastien Rodrigue, Vincent Burrus

**Affiliations:** Département de Biologie, Université de SherbrookeSherbrooke, QC, Canada

**Keywords:** *Vibrio cholerae*, cholera, antibiotic resistance, IncA/C, conjugative plasmid, pVCR94, *oriT*, SXT

## Abstract

Antibiotic resistance has grown steadily in *Vibrio cholerae* over the last few decades to become a major threat in countries affected by cholera. Multi-drug resistance (MDR) spreads among clinical and environmental *V. cholerae* strains by lateral gene transfer often mediated by integrative and conjugative elements (ICEs) of the SXT/R391 family. However, in a few reported but seemingly isolated cases, MDR in *V. cholerae* was shown to be associated with other self-transmissible genetic elements such as conjugative plasmids. IncA/C conjugative plasmids are often found associated with MDR in isolates of *Enterobacteriaceae*. To date, IncA/C plasmids have not been commonly found in *V. cholerae* or other species of *Vibrio*. Here we present a detailed analysis of pVCR94ΔX derived from pVCR94, a novel IncA/C conjugative plasmid identified in a *V. cholerae* clinical strain isolated during the 1994 Rwandan cholera outbreak. pVCR94 was found to confer resistance to sulfamethoxazole, trimethoprim, ampicillin, streptomycin, tetracycline, and chloramphenicol and to transfer at very high frequency. Sequence analysis revealed its mosaic nature as well as high similarity of the core genes responsible for transfer and maintenance with other IncA/C plasmids and ICEs of the SXT/R391 family. Although IncA/C plasmids are considered a major threat in antibiotics resistance, their basic biology has received little attention, mostly because of the difficulty to genetically manipulate these MDR conferring elements. Therefore, we developed a convenient derivative from pVCR94, pVCR94Δ X, a 120.5-kb conjugative plasmid which only codes for sulfamethoxazole resistance. Using pVCR94Δ X, we identified the origin of transfer (*oriT*) and discovered an essential gene for transfer, both located within the shared backbone, allowing for an annotation update of all IncA/C plasmids. pVCR94Δ X may be a useful model that will provide new insights on the basic biology of IncA/C conjugative plasmids.

## Introduction

Cholera is an infectious disease caused by the Gram-negative bacterium *Vibrio cholerae* that remains a major threat worldwide, especially for vulnerable territories where water supplies and sanitation are inadequate. The main symptoms of cholera are a profuse watery diarrhea and vomiting caused by the cholera toxin CtxAB encoded by CTXϕ, a filamentous phage carried by toxicogenic *V. cholerae* strains (Waldor and Mekalanos, 1996). The resulting rapid loss of fluids and electrolytes leads to severe and often lethal dehydration of patients within a few hours of onset, making oral antibiotics ineffective. Significant measures have been developed to fight this scourge, from public health improvement to vaccines and antibiotic therapies (Desai and Clemens, 2012; Harris et al., 2012). Since cholera is a self-limiting disease, patients usually recover rapidly with prompt and proper rehydration, and electrolyte replacement. Antimicrobial therapies may considerably reduce the severity of diarrhea and the duration of vibrio excretion yet increase the emergence and development of antibiotic resistance (Harris et al., 2012).

Currently, major mediators of antibiotic resistance in epidemic strains of *V. cholerae* remain Integrative and Conjugative Elements (ICEs), and mobile and chromosomal integrons (Waldor et al., 1996; Mazel et al., 1998; Hochhut et al., 2001; Burrus et al., 2006a; Mazel, 2006; Pugliese et al., 2009; Wozniak et al., 2009; Stalder et al., 2012). In fact, most epidemic strains recovered in the past 20 years harbor an ICE belonging to the SXT/R391 family (Burrus et al., 2006a; Reimer et al., 2011; Garriss and Burrus, 2013). The characterization of the first multi-drug resistant O139 *V. cholerae* epidemic strain isolated in India in the early 1990s led to the identification of SXT^MO10^, which was responsible for the resistance to sulfamethoxazole and trimethoprim (co-trimoxazole), chloramphenicol, and streptomycin of this isolate (Waldor et al., 1996). Nowadays, SXT-like relatives are found in most recent *V. cholerae* isolates including O1 strains such as the ones that caused the 2010 cholera epidemic in Haiti (Ceccarelli et al., 2011; Chin et al., 2011; Reimer et al., 2011; Sjolund-Karlsson et al., 2011; Katz et al., 2013). SXT, as well as all related ICEs, spread into and between bacterial populations *via* conjugation, and ensure their vertical inheritance into daughter cells during cell division by integrating into the host chromosome. In addition to resistance to several antibiotics, some members of the SXT/R391 ICEs also encode diguanylate cyclases that may facilitate their dissemination through manipulating the intracellular level of the second messenger c-di-GMP (Bordeleau et al., 2010).

A few *V. cholerae* epidemic surveys report the implication of conjugative plasmids in multi-drug resistance (MDR) (Sundaram and Murthy, 1984; Garrigue et al., 1986; Kruse et al., 1995; Dalsgaard et al., 2000a; Ceccarelli et al., 2006; Pugliese et al., 2009). Recent characterization of MDR in pandemic *V. cholerae* in Eastern China has shown that resistance to co-trimoxazole, ampicillin, streptomycin, gentamicin, tetracycline, and chloramphenicol was conferred by the large conjugative plasmid pMRV150, which belongs to the A/C incompatibility group (IncA/C) (Pan et al., 2008). Strikingly, while O139 epidemic strains isolated before 1997 were devoid of pMRV150, the proportion of isolates harboring pMRV150 or a related plasmid gradually rose to 92% between 2000 and 2006. IncA/C elements are large (>100 kb), broad host-range and single-copy conjugative plasmids that are mainly known for their considerable contribution to MDR phenotypes in pathogenic *Enterobacteriaceae* infecting humans, food products and food-producing animals (Welch et al., 2007; Fernandez-Alarcon et al., 2011). Comparative genomics studies revealed that the majority of the fully or partially sequenced IncA/C plasmids share a highly conserved backbone of genes of nearly 110 kb, coding for the conjugative transfer and replication machinery (Welch et al., 2007; Fricke et al., 2009; Fernandez-Alarcon et al., 2011). Interestingly, every predicted transfer gene encoded by the IncA/C plasmids are found in synteny in SXT/R391 ICEs, and the identities of these predicted protein sequences vary from 34 to 78% (Wozniak et al., 2009). Despite their prevalence in a wide range of pathogens isolated from very diverse geographic areas and their importance in the spread of MDR, characterization of IncA/C plasmids has been mostly epidemiological and remained limited to typing (Giske et al., 2012), antibiotic resistance profiling (Glenn et al., 2013), sequencing and comparative genomics (Welch et al., 2007; Fricke et al., 2009; Fernandez-Alarcon et al., 2011). Transcriptome analysis of the self-transferrable IncA/C plasmid pAR060302 revealed that most genes remain silent in laboratory conditions with the exception of a toxin/antitoxin gene locus and resistance genes, which seem to be constitutively transcribed (Lang et al., 2012). Interestingly, while little is known regarding their basic biology, IncA/C plasmids have been shown to specifically drive the *trans*-mobilization of the MDR-conferring *Salmonella* genomic island 1 (SGI1) of *S. enterica* (Doublet et al., 2005; Douard et al., 2010).

Rwanda was free of the seventh pandemic of cholera until 1978, when the disease started to be endemic in the African Great Lakes region. In years of political stability, cholera outbreaks in this region were shown to be influenced by climatic conditions, rainfall and fluctuation in plankton populations in what is known as the “cholera paradigm” (Bompangue Nkoko et al., 2011). However, from the 6th of April to the 4th of July 1994, unrest in the population led to a nationwide genocide, and to the exile of an estimated 8 00 000 refugees to the north of Goma, Democratic Republic of the Congo. Lack of an adequate and timely response, size of refugee camps, promiscuity and poor treatment in the early days led to a devastating cholera outbreak in the refugee population, affecting an estimated 36 000 individuals. Treatment was limited to oral rehydration in the early days of the outbreak. Isolated strains were shown to belong to the *V. cholerae* O1 El Tor Ogawa serotype, and were resistant to tetracyclines, aminopenicillins, co-trimoxazole, and nifuroxazide (Bioforce, 1996). An increase in resistance to nalidixic acid over the course of the outbreak was also observed, leaving few options for treatment (Cavallo et al., 1995). At the time, lack of data did not allow precise identification of determinants involved in antibiotic resistance.

In this study, we report the characterization of the novel IncA/C plasmid pVCR94, which is responsible for MDR of a Rwandan epidemic *V. cholerae* isolate. Sequence analysis of pVCR94 revealed its mosaic nature as well as the high degree of similarity of the structural genes responsible for transfer and maintenance among other IncA/C plasmids such as pIP1202 from *Y. pestis*. Transfer assays showed that pVCR94 is capable of mobilizing resistance determinants between *V. cholerae* strains as well as to and from *E. coli*. Genetic engineering of pVCR94 allowed us to initiate a more mechanistic study of this prototype IncA/C plasmid, in which we experimentally identified the location of the origin of transfer (*oriT*) together with a novel gene required for conjugative transfer, both within their shared backbone, allowing for an annotation update of all IncA/C plasmids.

## Materials and methods

### Bacterial strains and media

The bacterial strains and plasmids used in this study are described in Table 1. The strains were routinely grown in Luria-Bertani (LB) broth at 37°C in an orbital shaker/incubator and were preserved at −80°C in LB broth containing 15% (vol/vol) glycerol. For *E. coli*, antibiotics were used at the following concentrations: ampicillin (Ap), 100 μg/ml; chloramphenicol (Cm), 20 μg/ml; erythromycin, 200 μg/ml; gentamycin (Gn), 10 μg/ml; kanamycin (Kn), 50 μg/ml; rifampicin (Rf), 50 μg/ml; spectinomycin (Sp), 50 μg/ml; streptomycin, 200 μg/ml; sulfamethoxazole (Su), 160 μg/ml; tetracycline (Tc), 12 μg/ml; and trimethoprim (Tm), 32 μg/ml. For *V. cholerae*, antibiotics were used at the following concentrations: chloramphenicol, 2 μg/ml; kanamycin, 30 μg/ml; streptomycin, 10 μg/ml; tetracycline, 10 μg/ml. When required, bacterial cultures were supplemented with 0.3 mM DL-2, 6-diaminopimelic acid (DAP) or 0.02% L-arabinose. Antibiotics susceptibility profiling were done in three independent experiments using broth microdilution tests (Jorgensen and Ferraro, 2009).

**Table 1:** **List of strains and plasmids used in this study**.

**Strain or plasmid**	**Relevant genotype or phenotype**	**Source or Reference**
***E. coli***		
BW25113	F-, Δ (*araD-araB*)*567*, Δ *lacZ4787*(*::rrnB-3*), *λ^−^*, *rph-1*, Δ *(rhaD-rhaB)568*, *hsdR514*	(Datsenko and Wanner, 2000)
VB45	BW25113 Δ *recA*::*aad7* (Sp^R^)	This study
GG56	BW25113 Nx^R^	G. Garriss
CAG18439	MG1655 *lacZU118 lacI42*::Tn*10* (Tc^R^)	(Singer et al., 1989)
KB1	MG1655 *recA56 gutA52 gutR*::Tn*10*	Bettenbrock, unpublished
VB111	MG1655 Nx^R^	(Ceccarelli et al., 2008)
VB112	MG1655 Rf^R^	(Ceccarelli et al., 2008)
DPL12	VB111 Δ *dapA*::(*erm*-*pir*) (Nx^R^ Em^R^)	D. Poulin-Laprade
NC261	GG56 pVCR94 (Nx^R^ Su^R^ Tm^R^ Cm^R^ Ap^R^ Tc^R^ Sm^R^)	This study
VB557	GG56 pVCR94Δ X (Nx^R^ Su^R^)	This study
NC207	VB112 pVCR94 (Rf^R^ Su^R^ Tm^R^ Cm^R^ Ap^R^ Tc^R^ Sm^R^)	This study
NC213	VB111 pVCR94 (Nx^R^ Su^R^ Tm^R^ Cm^R^ Ap^R^ Tc^R^ Sm^R^)	This study
NC222	VB112 pVCR94Δ X (Rf^R^ Su^R^)	This study
NC367	VB111 pVCR94Δ X (Nx^R^ Su^R^)	This study
NC208	DPL12 pVCR94 (Nx^R^ Em^R^ Su^R^ Tm^R^ Cm^R^ Ap^R^ Tc^R^ Sm^R^)	This study
MS1	GG56 [pVCR94Δ X Δ *oriT*_1_::*aad7*] (Nx^R^ Su^R^ Sp^R^)	This study
MS2	GG56 [pVCR94Δ X Δ *vcrx062*::*aad7*] (Nx^R^ Su^R^ Sp^R^)	This study
MS3	GG56 [pVCR94Δ X Δ *vcrx001*::*aad7*] (Nx^R^ Su^R^ Sp^R^)	This study
MS4	VB45 pVCR94Δ X (Sp^R^ Su^R^)	This study
MS5	GG56 [pVCR94Δ X Δ *oriT*_2_::*aad7*] (Nx^R^ Su^R^ Sp^R^)	This study
MS6	GG56 [pVCR94Δ X Δ (*oriT*_2_-*vcrx001*)::*aad7*] (Nx^R^ Su^R^ Sp^R^)	This study
***V. cholerae***		
N16961	O1 El Tor strain (Sm^R^)	(Heidelberg et al., 2000)
E4	O1 El Tor strain; E7946 derivative Δ *ctxABN4* (Sm^R^ Kn^R^)	(Goldberg and Mekalanos, 1986)
F1939	Su^R^ Tm^R^ O1 El Tor 1994 clinical isolate from Rwanda	(O'shea et al., 2004b)
BI144	Su^R^ Tm^R^ exconjugant of F1939 × E4	M.K. Waldor
NC212	N16961 pVCR94 (Su^R^ Tm^R^ Cm^R^ Ap^R^ Tc^R^ Sm^R^)	This study
**PLASMIDS**		
pACYC177	Ap^R^ Kn^R^	New England Biolabs (Chang and Cohen, 1978)
pSIM5	pSC101 *cI857*; *P*_L_-*gam*-*bet*-*exo*; *cat*; Red expression vector (Ts, Cm^R^)	(Datta et al., 2006)
pMS1	pSIM5 Δ *cat*::*gen*; λRed expression vector (Ts, Gn^R^)	This study
pVI36	Sp^R^ PCR template for one-step chromosomal gene inactivation	(Ceccarelli et al., 2008)
pMA1	pACYC177 ‘*bla*::*oriT*(Kn^R^)	This study
pMA2	pACYC177 ‘*bla*::*vcrx062* (Kn^R^)	This study
pMA5	pACYC177 ‘*bla*::*oriT*-*vcrx001*(Kn^R^)	This study

Ap^R^, ampicillin resistant; Cm^R^, chloramphenicol resistant; Em^R^, erythromycin resistant; Gn^R^, gentamycin resistant; Kn^R^, kanamycin resistant; Nx^R^, nalidixic acid resistant; Rf^R^, rifampicin resistant; Su^R^, sulfamethoxazole resistant; Sm^R^, streptomycin resistant; Sp^R^, spectinomycin resistant; Tc^R^, tetracycline resistant; Tm^R^, trimethoprim resistant; Ts, thermosensitive.

### Bacterial conjugation assays

Conjugation assays were performed by mixing equal volumes of each donor and recipient strains that were grown overnight at 37°C. The cells were harvested by centrifugation for 3 min at 1200 g, washed in 1 volume of LB broth and resuspended in 1/20 volume of LB broth. Mating mixtures were then deposited on LB agar plates and incubated at 37°C for 6 h. The cells were recovered from the plates in 1 ml of LB broth and serially diluted before plating. Donors, recipients and exconjugants were selected on LB agar plates containing appropriate antibiotics.

### Molecular biology methods

Plasmid DNA was prepared using the EZ-10 Spin Column Plasmid DNA Minipreps Kit (Biobasic) according to manufacturer's instructions. All the enzymes used in this study were purchased from New England BioLabs. PCR assays were performed with the primers described in Table 2. The PCR conditions were as follows: (i) 3 min at 94°C; (ii) 30 cycles of 30 s at 94°C, 30 s at the appropriate annealing temperature, and 1 min/kb at 68°C; and (iii) 5 min at 68°C. When necessary, PCR products were purified using a EZ-10 Spin Column PCR Products Purification Kit (Biobasic) according to manufacturer's instructions. *E. coli* was transformed by electroporation as described by Dower et al. (1988) in a BioRad GenePulser Xcell apparatus set at 25 μF, 200 V and 1.8 kV using 1-mm gap electroporation cuvettes. Sequencing reactions were performed by the Plateforme de Séquençage et de Génotypage du Centre de Recherche du CHUL (Québec, QC, Canada).

**Table 2:** **DNA sequences of oligonucleotides used in this study**.

**Primer name**	**Nucleotide sequence (5′ to 3′)^a^**
vcr94W1F	TTAACTGCACATTCGGGATATTTCTCTATATTCGCGGTGTAGGCTGGAGCTGCTTCG
vcr94W1R	TAGAATAAGCCTCGATATAGTCATGTGACTAAAAGGATTCCGGGGATCCGTCGACC
oriT94_1WF	ATAGGCTCAGATAAACAGACCTTACCCTCGCATCGAGTGTAGGCTGGAGCTGCTTCG
oriT94_2WF	GAAATCCTCCAAAGATTGCTTTTAGATTGCTTTTCGGTGTAGGCTGGAGCTGCTTCG
oriT94_1WR	AATCTAGTTCTGTCACTAGGCTAACCCATCTTTGGAATTCCGGGGATCCGTCGACC
oriT94old_WF	TGTGTGACAAGAAGTATAGAGATTACGAGGTAGCCAGTGTAGGCTGGAGCTGCTTC
oriT94old_WR	GGATAGTTCTCCTGGATGGGAAGAAAGCCACAGTGACTGTCAAACATGAGAATTAA
oriT294Pr1_WF	TATCCACATTTCCTGTGCATAAACGGGGTTTTGGTAGTGTAGGCTGGAGCTGCTTC
oriT294Pr4_WR	TGATACTCGTCCTGTTCGGCCTTGCATACGAGACTTCTGTCAAACATGAGAATTAA
mobI94_WF	TAGGAATTGGATAGGAATTGGGAGGGTATTGAGGTGATTCCGGGGATCCGTCGACC
mobI94_WR	AATTCAGTGGCCGCTACAGATGCTGTCATGTTGTCAGTGTAGGCTGGAGCTGCTTCG
mobI94_2WR	AATTCAGTGGCCGCTACAGATGCTGTCATGTTGTCAATTCCGGGGATCCGTCGACC
recAWF	GACTATCCGGTATTACCCGGCATGACAGGAGTAAAAGTGTAGGCTGGAGCTGCTTCG
recAWR	GCCGCAGATGCGACCCTTGTGTATCAAACAAGACGAATTCCGGGGATCCGTCGACC
oriT144_F	NNNNNNGGATCCAAATTGACTCATGAAATCCTCCAA
oriT144_R	NNNNNNCTGCAGCCATCGCTCTGTTGGTAGACTCA
oriT94sec_F	GTATCAGGATCCGTATATGGGAACGCTGCACG
oriT94sec_R	AGACATCTGCAGGTGATACTCGTCCTGTTCG
oriT94oldBamHI_F	GTATCAGGATCCGACCCAGTAAGAGCAGAGC
oriT94oldPstI_R	AGTGACCTGCAGCACGGCTTCTTCATAGTCGG
oriTmobIPstI_R	AGACATCTGCAGTCACACCTCGTCGCTATGTG
Gen153F	AAGCTGTCAAACATGAGAATTCGAGCTCGGTA
Gen153(2)R	NNNNNNCCATGGGCCTTGAACGAATTGTTAG

a Restriction sites are underlined.

### Plasmids construction

Plasmids and oligonucleotides used in this study are listed in Tables 1, 2, respectively. λRed recombination encoding plasmid pMS1 was obtained after amplification of the gentamycin resistance cassette of pAH153 (Haldimann and Wanner, 2001) using primer pair Gen153F/Gen153(2)R, digestion of the resulting fragment using EcoRI and NcoI, and subsequent cloning into pSIM5 (Datta et al., 2006) digested with the same enzymes.

Plasmids used for complementation and mobilization assays containing *oriT* (pMA1), *oriT*-*vcrx001* (pMA5), and *vcrx062* (pMA2) were constructed in pACYC177. Fragments to be cloned were amplified using primers oriT144_F/oriT144_R (*oriT*), oriT144_F/oriTmobIPstI_R (*oriT-vcrx001*), and oriT94oldBamHI_F/oriT94oldPstI_R (*vcrx062*), respectively. Both inserts and vector were digested with BamHI and PstI, and ligated together by T4 DNA ligase reaction. Resulting plasmids were verified using restriction profiles and sequencing.

### Construction of chromosomal deletions and targeted deletions in pVCR94

Deletion mutants were constructed using the one-step chromosomal gene inactivation technique (Datsenko and Wanner, 2000). All deletions were designed to be non-polar. Primers used are listed in Table 2. The Δ*recA*::*aad7* mutation of *E. coli* VB45 was introduced in *E. coli* BW25113 using primer pair recAWF/recAWR and pVI36 as a template. Deletion of the MDR region located between *vcrx028* and *vcrx029* of pVCR94 was constructed in *E. coli* VB261 using primer pair vcr94W1F/vcr94W1R and pVI36 as template. The λRed recombination system was expressed using pMS1 as described for the pSIM expression vectors (Datta et al., 2006). The Sp^R^ cassette was removed from the resulting construction (pVCR94Δ X::*aad7*) by Flp-catalyzed excision using the pCP20 vector (Cherepanov and Wackernagel, 1995). The resulting strain containing pVCR94Δ X (VB557) was used for subsequent deletions of *oriT*_1_, *oriT*_2_, *vcrx062*, *vcrx001*, and *oriT*_2_-*vcrx001* using primer pairs oriT94_1WF/oriT94_1WR, oriT94_2WF/oriT94_1WR, oriT94old_WF/oriT94old_WR, mobI94_WF/mobI94_WR, and oriT94_2WF/mobI94_2WR, respectively. pVI36 and pSIM5 were used for all these constructions. All deletions were verified by PCR and antibiotic resistance profiling.

### Sequencing and sequence assembly of pVCR94ΔX

Using genomic DNA of *E. coli* MG1655 Rf^R^harboring pVCR94 or pVCR94Δ X, Illumina sequencing libraries were prepared as previously described (Rodrigue et al., 2010), and sequenced using 100-bp paired-end reads. The resulting sequences were assembled using version 2.6 of the *de novo* gsAssembler (Newbler) software by first removing sequences fully mapping to the *E. coli* MG1655 genome. The contigs were combined by using PCR and Sanger sequencing reactions. The resulting pVCR94Δ X sequence was annotated using the RAST automated pipeline (Aziz et al., 2008), manually curated and submitted to Genbank under accession KF551948.

### Blast atlas representation of sequences comparison

A circular BLAST Atlas was computed by GView (Petkau et al., 2010) on the GView server (https://server.gview.ca/) for each sequenced IncA/C plasmid using the BlastN algorithm and mapped against pVCR94Δ X. Sequences were aligned using raw sequence data, with an expect value cutoff of 1 × 10^−10^, an alignment length cutoff of 100, and a percent identity cutoff of 75%. Accession number of the sequences are as follow: pVmi603 (ACYU01000017.1), pIP1202 (NC_009141.1), pYR1 (CP000602.1), pRA1 (NC_012885.1), pR148 (NC_019380.1), pR55 (NC_016976.1), pNDM-KN (NC_019153.1), LS6 (JX442976.1), pNDM100469 (JN861072.1), pKPHS3 (CP003225.1), pSN254 (CP000604.1), pSD_174 (JF267651.1), pAM04528 (NC_012693.1), pPG010208 (NC_019065.1), pAPEC1990_61 (NC_019066.1), pAR060302 (NC_019064.1), pUMNK88 (NC_017645.1), pNDM-1_Dok01 (NC_018994.1), pNDM10505 (JF503991.1), pNDM102337 (JF714412.2), pEH4H (NC_012690.1), pMR2011 (JN687470.1), pTC2 (JQ824049.1), pP99-018 (AB277723.1), pP91278 (AB277724.1), pXNC1 (FN667743.1).

## Results

### The 1994 rwandan cholera outbreak involved a multiresistant *V. cholerae* isolate harboring a conjugative plasmid

*V. cholerae* F1939 is a co-trimoxazole-resistant O1 El Tor clinical isolate recovered from a refugee camp during the 1994 Rwanda cholera outbreak (O'shea et al., 2004a,b). F1939 was initially identified as a strain capable of transferring the co-trimoxazole resistance to *V. cholerae* E4 (Table 1). As such, we expected that the resulting exconjugant, *V. cholerae* BI144, would carry an ICE of the SXT/R391 family, which are major vectors of co-trimoxazole resistance in epidemic *V. cholerae* strains. All our attempts to PCR amplify markers typical of SXT/R391 ICEs (*int*_SXT_ and *setR*) from genomic DNA of BI144 failed (data not shown). Instead, plasmid typing based on PCR amplification of *repA* revealed that in the exconjugant *V. cholerae* BI144 co-trimoxazole resistance was conferred by a plasmid of the IncA/C group that we named pVCR94 (plasmid *Vibrio cholerae* Rwanda 1994).

### pVCR94 disseminates multidrug resistance at high frequencies

To further characterize pVCR94, mating experiments were carried out to transfer the plasmid from *V. cholerae* BI144 to *E. coli* VB111 and VB112 strains (MG1655 Nx^R^ and MG1655 Rf^R^, respectively) by co-trimoxazole selection. As expected, since IncA/C plasmids have a broad host-range, pVCR94 efficiently transferred to and was stably maintained in *E. coli*, giving rise to NC213 and NC207, respectively. Additional antibiotic resistance testing of *E. coli* exconjugants revealed that pVCR94 also confers resistance to ampicillin, streptomycin, tetracycline, and chloramphenicol, but not to gentamycin, kanamycin, rifampicin, nalidixic acid, and erythromycin (Table 3).

**Table 3:** **Minimal inhibitory concentrations (MIC) of 12 antibiotics against *E. coli* carrying pVCR94 or its Δ X mutant**.

	**VB111**		**NC213 (VB111 pVCR94)**		**NC367 (VB111 pVCR94Δ X)**	
**Antibiotic**	**MIC (μg/mL)^a^**	**Phenotype^b^**	**MIC (μ/mL)^a^**	**Phenotype^b^**	**MIC (μg/mL)^a^**	**Phenotype^b^**
Ampicillin	<3.125	S	>3 200	R	<3.125	S
Chloramphenicol	5	S	320	R	10	S
Erythromycin	100	S	100	S	100	S
Gentamycin	5	S	2.5	S	5	S
Kanamycin	6.25	S	6.25	S	6.25	S
Nalidixic acid^c^	<5	S	<5	S	nd	S
Rifampicin	nd	S	nd	S	nd	S
Streptomycin	12.5	S	>6 400	R	12.5	S
Spectinomycin	25	S	25	S	25	S
Sulfamethoxazole	nd	S	1 280	R	1 280	R
Tetracycline	3	S	384	R	3	S
Trimethoprim	<1	S	1 024	R	<1	S

a nd, not determined, test was only done on solid agar plate. All assays were carried out in three independent replicates.

b R, resistant; S, susceptible.

c These assays were carried out using the Rf^R^ derivatives VB112, NC207 (VB112 pVCR94) and NC222 (VB112 pVCR94Δ X), respectively.

**Figure 1:**
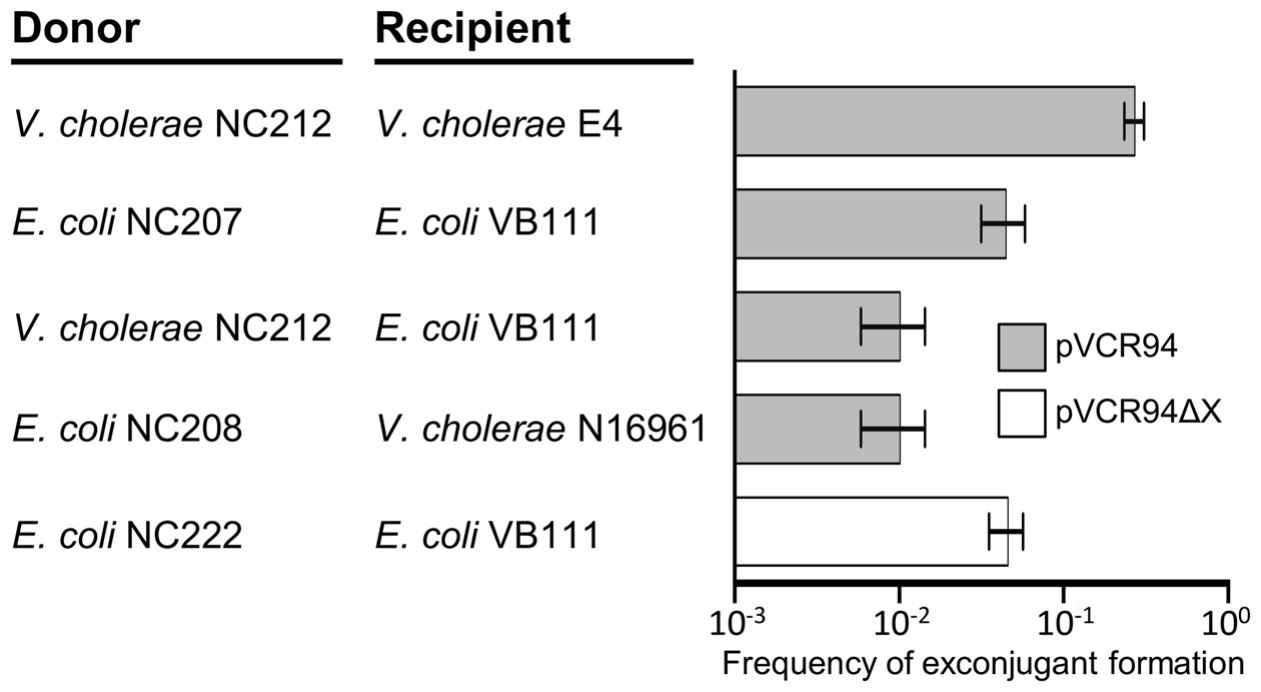
**Conjugative transfer of pVCR94 and the pVCR94ΔX mutant**. *V. cholerae* intraspecific transfers were carried out using N16961 (Sm^R^) containing pVCR94 (Su^R^ Tm^R^ Cm^R^ Ap^R^ Tc^R^ Sm^R^) (NC212) as the donor and E4 (Sm^R^ Kn^R^) as the recipient. Exconjugants were selected as Kn^R^ Tc^R^ Cm^R^ colonies. For *E. coli* intraspecific transfers, the donor (pVCR94^+^ or pVCR94Δ X^+^) and recipient were Rf^R^ and Nx^R^ derivatives of MG1655, respectively. Exconjugants were selected as Nx^R^ Tc^R^ Cm^R^ (pVCR94) or Nx^R^ Su^R^ (pVCR94Δ X) colonies. For interspecific transfers, on one hand, *V. cholerae* N16961 (Sm^R^) containing pVCR94 was used as the donor, *E. coli* MG1655 Nx^R^ (VB111) as the recipient and exconjugants were selected as Nx^R^ Tc^R^ Cm^R^ resistant colonies. On the other hand, *E. coli* NC208, an otherwise isogenic strain auxotrophic for diaminopimelic acid (DAP) and containing pVCR94, was used as the donor, the Sm^R^ strain *V. cholerae* N16961 as the recipient and *V. cholerae* exconjugants were selected as Sm^R^ Tc^R^ Cm^R^ colonies in the absence of DAP. The bars represent the mean and standard deviation values obtained from at least three independent experiments. pVCR94 and pVCR94Δ X transfer frequencies are expressed as a number exconjugant per recipient colonies.

Preliminary tests showed that pVCR94 self-transfers at high frequency. To evaluate its potential to disseminate MDR, tetracycline and chloramphenicol resistance markers were used to test conjugative transfer of pVCR94 among and between *E. coli* and *V. cholerae*. Intraspecific transfer of pVCR94 was first tested from its primary host *V. cholerae*. Two El Tor variants were used, NC212 (N16961 containing pVCR94) as the donor and E4 as the recipient, revealing that pVCR94 transfers at very high frequency (~3 × 10^−1^ exconjugant per recipient) (Figure 1). For intraspecific transfer of pVCR94 in *E. coli*, otherwise isogenic Nx^R^ and Rf^R^ MG1655 derivative strains VB111 and VB112 were used. Despite a ~6-fold reduction of transfer, pVCR94 still transferred very efficiently under these conditions (Figure 1). Interspecific transfer of pVCR94 using *V. cholerae* NC212 as a donor and *E. coli* VB111 as a recipient and *vice versa*, *E. coli* NC208 as a donor and *V. cholerae* N16961 as a recipient, occurred at roughly the same frequency (~10^−2^ exconjugant per recipient cell) (Figure 1).

### pVCR94 shares a common backbone with IncA/C plasmids

**Figure 2:**
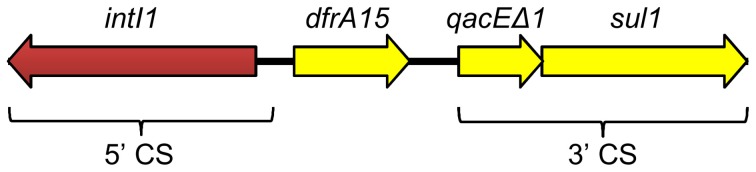
**Schematic representation of the class 1 integron located near to the *sul2* gene of pVCR94**. The *dfrA15* cassette confers resistance to trimethoprim. The location of the 5′- and 3′ conserved sequences (CS) typical of class 1 integrons are indicated at the bottom.

**Figure 3:**
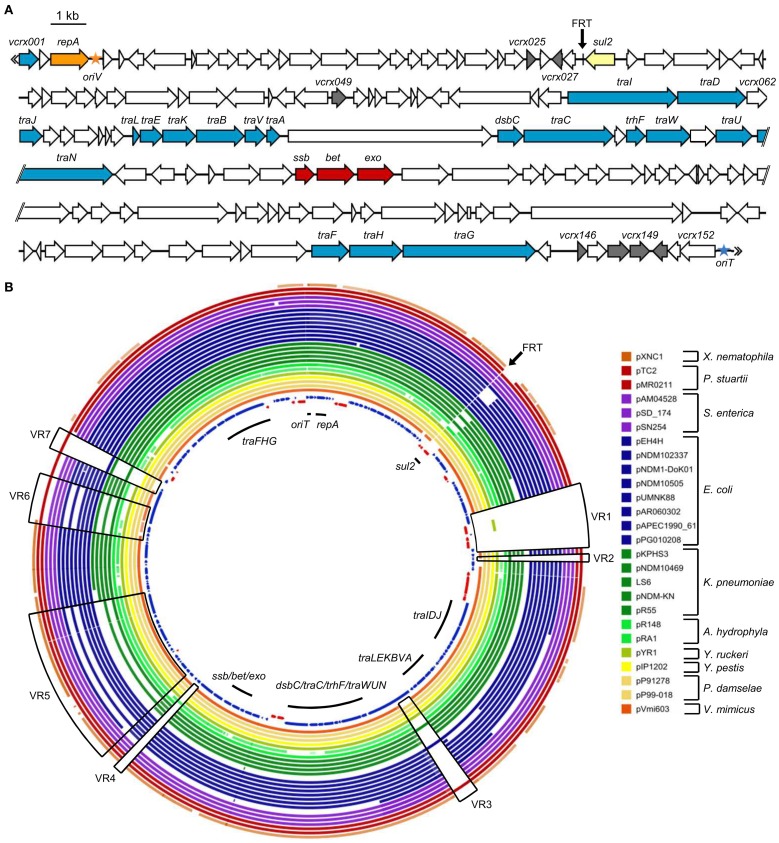
**Sequence analysis of pVCR94Δ X. (A)** Schematic representation of the genetic organization of pVCR94Δ X. The location and orientation of ORFs are indicated by arrowed boxes. The color of the arrowed boxes depicts the putative function or relationships of each ORF deduced from functional analyses and BLAST comparisons: white, unknown function; blue, conjugative transfer; orange, replication; yellow, antibiotic resistance; gray, regulation; red, homologous recombination. The origin of replication (*oriV*) and the origin of transfer (*oriT*) are symbolized by an orange and a blue star, respectively. The position of the scar resulting from the deletion of the multidrug resistance gene cluster is indicated (FRT site). **(B)** Genetic comparison of pVCR94Δ X and other sequenced IncA/C plasmids. A BLAST Atlas was constructed with the pVCR94Δ X sequence set as the reference (innermost circle). All completely sequenced IncA/C plasmids available in Genbank were aligned according to their raw sequence data toward pVCR94Δ X using a BlastN algorithm. Coding sequences of pVCR94Δ X appear on the innermost circle in blue for the positive strand, and red for the negative strand. All other aligned plasmids sequences are represented only according to their sequence homology toward the reference. Full color saturation represents 100% sequence identity, and gaps indicate regions of divergence (<75% percentage of nucleic acid identity). The black arrow indicates the position of the deletion that generated pVCR94Δ X. Part of an IncA/C plasmid closely related to pVCR94 was detected among at least 3 different contigs of the unassembled *V. cholerae* RC9 genome (Genbank accession number ACHX00000000). Since the sequence of this plasmid is not assembled and probably not complete, it was not included in this analysis.

Genomic DNA of *E. coli* NC207 (VB112 harboring pVCR94) was extracted to sequence the plasmid using the Illumina technology. A draft assembly generated a sequence of 134,484 bp. Initial sequence analyses revealed the presence of only three resistance genes, *sul1*, *sul2*, and *dfrA15*, conferring resistance to co-trimoxazole. *sul1* and *dfrA15* belong to a class 1 mobile integron devoid of any other integron cassette (Figure 2) Identical integron structures have already been described in clinical *V. cholerae* strains belonging to non-O1, non-O139 serogroups isolated in the mid-90s in Thailand and India (Dalsgaard et al., 2000b; Thungapathra et al., 2002). Further analysis confirmed that the sequence of pVCR94 was partial; a segment of unknown size overlapping most other antibiotic resistance genes could not be properly assembled. We constructed a deletion mutant, pVCR94Δ X, which lacks this region (see below), and submitted the resulting 120,572-bp sequence to Genbank database (accession number KF551948) (Figure 3A).

Comparative genomics confirmed the conservation in pVCR94Δ X of the large core set of genes that are common to all members of the IncA/C group (Figure 3B), including genes that may be involved in the regulation of IncA/C conjugative transfer (Table 4). Among them, *vcrx148* and *vcrx149* are particularly interesting. Pfam analyses (database v27.0) revealed that the Vcrx149 protein contains an FlhC signature (Pfam PF05280) whereas Vcrx148 has very weak homology with the FlhD domain (Pfam PF05247). Sequence comparisons also brought to light variable regions encoding hypothetical proteins of unknown function (VR2, VR3, VR4, VR5, and VR6) (Figure 3). The 4.5-kb variable region VR1 contains DNA that is only found in pVCR94 and codes for a putative cadmium-bromine pump efflux and its cognate regulator (*vcrx049*, Table 4) along with a transposase gene. The 1.5-kb VR7 region codes for a putative transmembrane protein with an EndoU_bacteria nuclease domain (Pfam PF14436) and is predicted to be a secreted bacterial toxin (Zhang et al., 2011).

**Table 4:** **Open reading frames (ORFs) of pVCR94ΔX coding for putative transcriptional regulators**.

**ORF name**	**Size (aa)^a^**	**Predicted function**	**Most significant Pfam matches**	**Conserved in IncA/C plasmids**	**Ortholog expressed in pAR060302^b^**
*vcrx025*	90	HUβ-like DNA-binding protein	Bac_DNA_binding (PF00216)	Yes	+
*vcrx027*	100	Cro-like transcriptional regulator (Xre)	HTH_37 (PF13744)	Yes	+++
*vcrx049*	135	Cd(II)/Pb(II)-responsive transcriptional regulator	MerR_1 (PF13411)	No	Absent
*vcrx146*	90	Ner-like DNA-binding protein	HTH_35 (PF13693)	Yes	0
*vcrx148*	202	FlhD-like transcriptional activator	FlhD (PF05247)^c^	Yes	0
*vcrx149*	183	FlhC-like transcriptional activator	FlhC (PF05280)	Yes	0
*vcrx150*	139	H-NS-like DNA-binding protein	Histone_HNS (PF00816)	Yes	++

a Size in amino-acids of the predicted translation product.

b Level of expression of ortholog genes in the IncA/C plasmid pAR060302 (Lang et al., 2012).

c This domain was reported as an insignificant Pfam-A match.

Interestingly, comparison of the genes found in variable regions of pVCR94 with those of other sequenced IncA/C plasmids indicate that pVCR94 is more closely related to plasmids recovered from *P. damselae* (pP91278 and pP99-018), *K. pneumoniae* (pR55), *E. coli* (pPG010208), and *P. stuartii* (pTC2) than from the plasmid identified in *V. mimicus* (pVmi603) (Figure 3).

### pVCR94ΔX, a convenient prototype for the study of the basic biology of IncA/C plasmids

To facilitate future studies of IncA/C plasmid biology without the challenges and limitations associated with the MDR phenotype usually conferred by these mobile elements, we decided to construct a mutant of pVCR94 coding for a reduced set of antibiotic resistance. Antibiotic resistance markers carried by vectors used for expression of λRed recombination system, Ap^R^ for pKD46 and Ap^R^, Cm^R^, Kn^R^, or Sp^R^ for pSIM vectors (Datsenko and Wanner, 2000; Datta et al., 2006), are not compatible with pVCR94 and most known IncA/C plasmids. To circumvent this problem, we constructed a Gn^R^ derivative of pSIM5 (pMS1) to allow expression of λRed recombination function in this multiple antibiotic resistance context.

Using pMS1 and a Sp^R^ cassette, we deleted the large fragment containing the MDR-conferring genes in pVCR94 and located between *vcrx028* and *vcrx029* (*sul2*), which encode a hypothetical protein and resistance to sulfamethoxazole respectively. After elimination of the Sp^R^ cassette, antibiotic resistance examination confirmed the sensitivity of *E. coli* MG1655 containing pVCR94Δ X (NC367) to all tested antibiotics but sulfamethoxazole (Table 3). Despite the large deletion, pVCR94Δ X was able to stably maintain in *E. coli*. Finally, mating experiments showed that pVCR94Δ X remains self-transmissible at the same frequency as wild-type pVCR94 (Figure 1). Although the exact gene content and size of the region that was deleted remains to be established, our functional tests indicate that pVCR94Δ X now constitutes a convenient prototype for in-depth molecular study of pVCR94 and related IncA/C plasmids.

### Identification of *oriT*_IncA/C_, the origin of transfer of IncA/C plasmids

**Figure 4:**
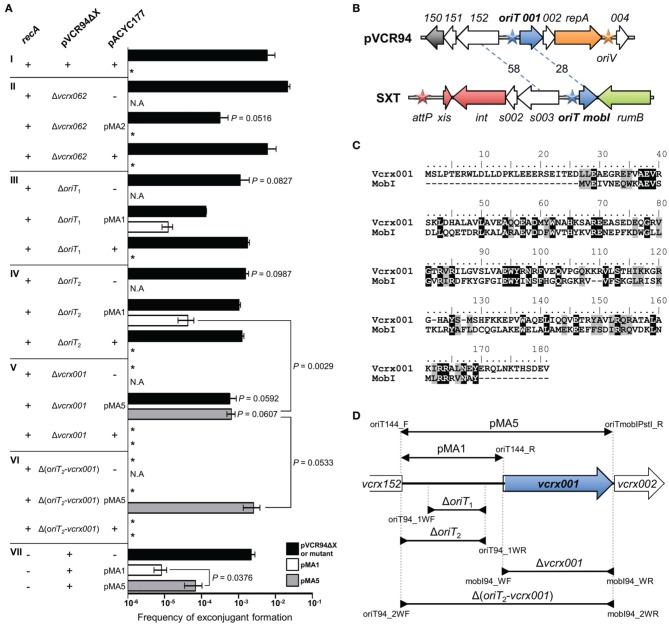
**Identification of the *oriT* locus of pVCR94. (A)** Conjugation and mobilization assays were carried out to assess the impact of deletion, and the ability to *trans*-initiate transfer of the indicated regions cloned into pACYC177 (see panel **D**). In every experiment, pVCR94Δ X was used as a positive control for efficiency of transfer, and pACYC177 was used as a negative control for mobilization assays. Frequency of transfer of each deleted region was compared to the mobilization frequency of its cognate cloned sequence. Within each mating experiment, exconjugants were selected for their acquisition of either the pACYC177 derivatives, pVCR94Δ X derivatives, or for cotransfer of both, when applicable. All mating experiments that involved *recA*^+^ strains were made from BW25113 Nx^R^ as donor toward the Tc^R^ strain CAG18439 as recipient. Transfers done in a *recA*^−^ background involved BW25113 Δ *recA*::*aad7* (VB45) and KB1 as donor and recipient strains, respectively. The bars represent the mean and standard deviation values obtained from three independent experiments. Asterisk indicates that frequency of exconjugant formation was below the limit of detection (<10^−8^). N.A. indicates that the selection was not applicable in the mating experiment. Statistical analyses were performed using the two-tailed Student's *t*-test. *P*-values are indicated above the bars when comparison referred to pVCR94Δ X (panel **AI**) or above the brackets comparing two bars. **(B)** Comparison of the genetic context of *oriT* loci in SXT and pVCR94. Arrows of similar color represent genes predicted to have similar functions: dark blue, conjugative transfer; orange, replication; gray, H-NS-like DNA-binding protein; red, site-specific recombination; green, DNA repair; white, unknown function. Blue stars indicate the *oriT* loci. The orange star indicates the position of the origin of replication (*oriV*) of pVCR94 based on identity with pRA1. The red star indicates the position of the *attP* site for chromosomal integration of SXT by site-specific recombination. The percent of identity of orthologous proteins are indicated on dashed lines. **(C)** Amino acid sequence alignment of the translation products of *vcrx001* and *mobI* computed by Clustal Omega (Sievers et al., 2011). Similarities (gray) and identities (black) are visualized using the BLOSUM62 substitution matrix. **(D)** Schematic representation of the region of pVCR94 encompassing the end of *vcrx152* and the end of *vcrx002*. The inserts of the plasmids used in mobilization experiments, all of which were derived from the low-copy non-mobilizable vector pACYC177, are represented above the genetic map by overlapping segments delimited by arrows pointing outwards. Deletions within the region are depicted below the genetic map by overlapping segments delimited by arrows pointing inwards. The positions of the oligonucleotides used for amplification and cloning or construction of the deletions are indicated (Table 2).

Conjugative transfer is initiated at a specific *cis*-acting locus called the origin of transfer (*oriT*) by a DNA relaxase, which is typically called TraI. Fricke et al. (2009) and Welch et al. (2007) proposed to position the *oriT* locus of IncA/C plasmids (*oriT*_IncA/C_) between the genes *traD* and *traJ*. This annotation was based not on experimental data but rather on an analogy with the location of *oriT* of the ICE SXT proposed by Beaber et al. (2002). However, the sequence located between *traD* and *traJ* in SXT has been shown to be unable to support the mobilization of a non-mobilizable plasmid and *oriT* of the SXT/R391 ICEs was subsequently relocated upstream of a gene named *mobI* (Ceccarelli et al., 2008). Therefore, we hypothesized that the region located between *traD* and *traJ* in IncA/C plasmids, which contains *vcrx062* in pVCR94Δ X, is not *oriT*_IncA/C_ and experimentally investigated the location of the *oriT*_IncA/C_ in our model. Noteworthy in the following experiments, the use of alternative donor and recipient strains caused a tenfold reduction in transfer of pVCR94Δ X compared to the previous experiments (Figures 1, 4A), thereby suggesting that the genetic background has a notable influence on the efficiency of IncA/C plasmids transfer.

First, we verified whether the region containing *vcrx062* was required for transfer and whether it was sufficient to support the mobilization of the non-mobilizable low-copy plasmid pACYC177. We used as a donor *E. coli* MS2, which harbors pVCR94ΔX Δ*vcrx062* (Sp^R^), to mobilize a pACYC177 derivative containing *vcrx062* (pMA2, Kn^R^) to the Tc^R^ strain *E. coli* CAG18439. Exconjugants were independently selected for acquisition of pMA2 (Tc^R^ Kn^R^) or pVCR94Δ X Δ *vcrx062* (Tc^R^ Sp^R^). We observed that deletion of *vcrx062* did not affect the transfer efficiency of pVCR94Δ X (Figures 4AI,II). Furthermore, the *vcrx062* locus was incapable to initiate transfer of pMA2 as no Tc^R^ Kn^R^ exconjugant could be recovered (Figure 4AII). These results provide convincing evidence that the locus located between *traD* and *traJ* is not an *oriT* for IncA/C plasmids. Interestingly, in the presence of pMA2, transfer of pVCR94Δ X dropped significantly. This phenotype was not observed in the presence of pACY177.

In the ICEs of the SXT/R391 family, *oriT* is located in a large intergenic region between two divergent genes: *mobI*, which is crucial for conjugative transfer, and *s003*, a gene of unknown function (Ceccarelli et al., 2008). Comparison of the *s003*-*mobI* locus of SXT with the corresponding region of pVCR94 revealed striking similarities in gene organization (Figure 4B). In fact, in pVCR94 *vcrx001* encodes a protein sharing a weak identity with MobI (Figure 4C) and *vcrx152* encodes a protein sharing 58% identity with S003. To test whether *oriT*_IncA/C_ was located between *vcrx152* and *vcr001*, plasmid pMA1 (Kn^R^) was constructed by cloning this intergenic region (putative *oriT*) into pACYC177 (Figure 4D). pMA1 was subsequently introduced into *E. coli* MS1 and MS5, two Sp^R^ strains containing pVCR94Δ X Δ *oriT*_1_ or Δ *oriT*_2_, respectively (Figure 4D). In both of these mutants, deletions were designed to preserve the promoter region upstream of *vcrx001*. We observed that while neither deletion abolished the transfer of pVCR94, both led to a significant 10-fold reduction of transfer (Figures 4AIII,IV). In addition, while mobilization of pACYC177 by pVCR94Δ X Δ *oriT*_1_ or Δ *oriT*_2_ was undetectable, pMA1 was mobilized at a frequency of 1.3 × 10^−5^ to 4.0 × 10^−5^ exconjugant per donor cell (Figures 4AIII,IV). Altogether, these results suggest the presence of an *oriT* between *vcrx152* and *vcrx001* in pVCR94Δ X as this locus is a weak yet suitable substrate for transfer initiation. However, the low efficiency of mobilization conferred by this locus and the ability of the Δ *oriT*_2_ mutant of pVCR94Δ X to transfer efficiently suggest that other alternative *oriT* loci may exist in IncA/C plasmids. Alternatively, the actual *oriT* may also include the promoter region of *vcrx001* and perhaps the 5′ end this open reading frame (see below).

### vcrx001 is an essential gene for conjugative transfer of IncA/C plasmids

Transfer of SXT is abolished in the absence of MobI, a protein that is likely an auxiliary component of the relaxosome (Ceccarelli et al., 2008). MobI of SXT/R391 ICEs exhibits 28% identity (40% similarity) with its IncA/C ortholog *vcrx001* (Figures 4B,C). Annotated in all available IncA/C plasmids sequences as a gene coding for a hypothetical protein, the importance of *vcrx001* in conjugative transfer of these plasmids has never been investigated. Furthermore, the low frequency of mobilization of pMA1 observed above could be due to the lack of an adjacent *mobI*-like gene as it has already been reported for SXT (Ceccarelli et al., 2008).

We constructed *E. coli* MS3, which harbors pVCR94Δ X Δ *vcrx001* (Sp^R^), to test the importance of the *mobI* ortholog for IncA/C transfer. As reported for SXT Δ *mobI*, transfer of the Δ *vcrx001* mutant of pVCR94Δ X was completely abolished (Figure 4AV), confirming the essential role of *vcrx001* in conjugative transfer. To further investigate its function, the 1063-bp fragment overlapping *vcrx001* and the upstream intergenic region was cloned into pACYC177 to generate pMA5 (Figure 4D). Mating experiments using pMA5 were carried out to evaluate the impact of the *cis*-expression of *vcrx001* on a mobilizable plasmid containing *oriT*_IncA/C_. Exconjugants colonies selected for transfer of pMA5 or pVCR94Δ X Δ *vcrx001* were recovered at a frequency of 1 × 10^−3^, which is not dramatically different from transfer of pVCR94Δ X (Figure 4AV). Thus, abolition of transfer observed in the Δ *vcrx001* mutant is not due to a polar effect of the deletion on expression of the *repA* gene, as it can be complemented by expression from its endogenous promoter on a plasmid. Furthermore, the presence of *vcrx001 in cis* significantly improved the transfer of pMA5 over pMA1 (10-fold increase).

Since pVCR94Δ X Δ *vcrx001* and pMA5 share an identical 514-bp fragment corresponding to the *vcrx152*-*vcrx001* intergenic region, we tested whether the mobilization observed for pMA5 could result from cointegrate formation between the two plasmids mediated by homologous recombination. We used two complementary approaches to test this hypothesis. First to rule out the RecA-mediated recombination, mobilization of pMA1 and pMA5 by pVCR94Δ X was tested using Δ *recA* donor and recipients strains (*E. coli* VB45 and KB1, respectively). Results showed that transfer of pVCR94Δ X is RecA-independent and that pMA5, as well as pMA1, were still mobilized in this *recA* background, with the same 10-fold improvement due to the presence of *vcxr001* in pMA5 (Figure 4AVII). Second, since homologous recombination could also potentially be mediated by the activity of the putative λRed recombination system carried by IncA/C plasmids (*ssb*, *bet*, *exo*, see Figure 3A) we constructed a Δ (*oriT*_2_-*vcrx001*) mutant of pVCR94Δ X devoid of homologous sequence in pMA5, thereby resulting in *E. coli* MS6 (Figure 4D). pVCR94Δ X Δ (*oriT*_2_-*vcrx001*) was no longer able to transfer and this mutation was not complemented by expression of *vcrx001* from pMA5, thereby confirming that *oriT*_IncA/C_ is located within the deleted fragment (Figure 4AVI). Furthermore pMA5 itself remained mobilizable at very high frequency by pVCR94Δ X Δ (*oriT*_2_-*vcrx001*), despite the absence of homologous sequences between the two plasmids confirming that the insert of this plasmid contains *oriT*_IncA/C_. Interestingly, mobilization of pMA5 was even improved in this context suggesting that different replicons carrying *oriT*_IncA/C_ likely compete against with each other during transfer.

## Discussion

Cholera remains one of the most devastating human diseases in the world mainly because of toxicigenicity, transmissibility, the rapid multiplication of *V. cholerae* in favorable conditions, and the MDR phenotype of pandemic and epidemic strains. In most modern cholera outbreaks, MDR has been shown to be conferred by SXT/R391 ICEs (for review, Garriss and Burrus, 2013). In the present study, we described the IncA/C plasmid pVCR94 recovered from the multidrug-resistant strain *V. cholerae* O1 El Tor F1939 as the element most likely responsible for the co-trimoxazole resistance during the severe 1994 cholera epidemics in Rwanda. This conclusion is further supported by the absence of the ICE SXT from two other co-trimoxazole resistant *V. cholerae* O1 El Tor isolates, F1873 and F1875, recovered from Rwandan refugees in the summer of 1994 in Goma, Democratic Republic of the Congo (Waldor et al., 1996). Mating assays showed that pVCR94 is a high-efficiency driver of antibiotic resistance dissemination between *V. cholerae* strains as well as to and from *E. coli*. Studies carried out on Asian pandemic isolates of *V. cholerae* underline the rapid emergence and dissemination of IncA/C plasmids as key drivers of antibiotic resistance between 1994 and 2000 (Pan et al., 2008). Although the rate of dissemination of SXT/R391 ICEs between vibrios is rather low in the laboratory as their transfer frequency rarely exceeds 1 × 10^−5^ in intra-species mating experiments (Waldor et al., 1996; Burrus et al., 2006b; Osorio et al., 2008), these elements have been extremely successful in invading environmental and clinical *V. cholerae* in the past two decades (Chin et al., 2011; Mutreja et al., 2011; Yu et al., 2012; Garriss and Burrus, 2013; Katz et al., 2013). On the contrary, IncA/C plasmids such as pVCR94 are much more efficient (1 × 10^−1^) and seem to transfer even better between vibrios than in *E. coli*. It has recently been shown that pVCR94 transfers at high frequency between *E. coli* strains in sludge resulting from the coagulation/flocculation treatment of surface water, reaching the highest frequency after 72 h with about 1 × 10^−2^ exconjugants per recipient cell (Pariseau et al., 2013). These observations indicate that, in laboratory conditions as well as in a simulated environmental setting, pVCR94 is a very efficient conjugative plasmid able to invade a significant proportion of the surrounding compatible cells. Knowing the prevalence of IncA/C plasmids in pathogenic bacteria isolated from humans and food-producing animals, their circulation in clinical and environmental *V. cholerae* isolates is worrisome and their impact on the emergence of new pathogenic isolates needs to be surveyed.

Until now, relatively little has been done to characterize the basic biology of IncA/C plasmids despite the significant threat that they represent in the war against MDR pathogenic bacteria. In fact, one of the major challenges encountered to genetically manipulate IncA/C plasmids is their propensity to confer MDR to their host. As a consequence, the majority of antibiotic resistance phenotypes conferred by molecular engineering tools (plasmids, selection gene cassettes) are also conferred by IncA/C plasmids. A gentamycin-resistant version of a λRed recombination expression plasmid that is compatible with pVCR94 allowed us to construct pVCR94Δ X, a derivative lacking the MDR region. Sequence analyses and experimental evidences revealed that pVCR94Δ X carries the core sequences necessary for self-transfer and maintenance of IncA/C plasmids. Using this plasmid as a convenient prototype for the study of IncA/C biology, we have identified the locus containing *oriT*_IncA/C_. Extensive protein sequence conservation and gene synteny between IncA/C plasmids and SXT/R391 ICEs were crucial in this process. We also showed that *vcrx001*, the ortholog of *mobI* of SXT, plays a key role in transfer and enhances the mobilization of a plasmid containing *oriT*_IncA/C_ when it is located on the same replicon. In SXT, *mobI* was reported to be a *cis*-acting sequence coding for a putative auxiliary component of the relaxosome required for SXT transfer (Ceccarelli et al., 2008). Surprisingly, deletion of the center (Δ *oriT*_1_) and the left part (Δ *oriT*_2_) of the intergenic region between *vcrx152* and *vcrx001* did not affect much the transfer of pVCR94. This suggests that *oriT*_IncA/C_ overlaps the right part of this intergenic region and perhaps the 5′ end of *vcrx001*. Experiments aimed at discovering the minimal *oriT* sequence required for initiation of transfer of IncA/C plasmids are ongoing. Furthermore, the mutation Δ *oriT*_2_ which removed the promoter upstream of *vcrx152* likely prevents expression of three genes, two of unknown function (*vcrx152* and *vcrx151*) and one coding for a predicted H-NS-like protein (*vcrx150*) (Table 2). H-NS-like proteins encoded by conjugative plasmids have been shown to provide stealth function helping the transmission of the plasmid into a naïve host (Doyle et al., 2007). While the ortholog of *vcrx150* was shown to be expressed in the IncA/C plasmid pAR060302 (Lang et al., 2012), our deletion did not seem to alter significantly the stability of pVCR94 or its ability to transfer.

Mobile genetic elements are characterized by a modular structure, each module containing the genes and sequences involved in a same biological function (Toussaint and Merlin, 2002). Clustering is an efficient way to exchange and transfer “en bloc” fully functional modules and thus confers new adaptive traits in one event (Lawrence and Roth, 1996). Previous sequence analyses of IncA/C plasmids highlighted their modular structure, with specific variable regions corresponding to mobile integrons and transposable elements conferring adaptive traits such as multiple antibiotics resistance (Welch et al., 2007; Fricke et al., 2009). As observed for pIP1202 and other IncA/C plasmids, pVCR94 carries a class 1 integron. While this integron carries only a *dfrA15* cassette conferring resistance to trimethoprim, its impact should not be underestimated. On one hand, Baharoglu et al. (2010) demonstrated that incoming single-stranded DNA during conjugative transfer triggers the SOS response in the recipient cell, in both *E. coli* and *V. cholerae*. On the other hand, SOS response enhances integron cassette rearrangements through excision/integration, providing opportunities for different integrons present in the same host to exchange cassettes (Guerin et al., 2009). Thus, conjugative transfer of pVCR94 could lead to integron cassettes trapping and drive their dissemination between bacterial communities. Sequence analysis of pVCR94 also revealed the specific region VR1 corresponding to a putative transposon that could confer resistance to heavy metals. This resistance cluster is exclusively carried by the chromosome of various Gram-negative bacteria. Thus, IncA/C plasmids drive horizontal transfer of chromosomal loci and other mobile genetic elements at high frequency by *cis*-mobilization. Beyond intra- and inter-molecular rearrangements among and between chromosome and plasmid, pVCR94 could also mediate *trans*-mobilization of genomic islands present in its host range, as demonstrated for the *S. enterica* pathogenicity island SGI1 (Douard et al., 2010). Finally, two core genes of IncA/C plasmids code for homologs of λBet and λExo proteins of the λRed recombination system. Many conjugative plasmids and ICEs code for such proteins, which have been shown to generate diversity of SXT/R391 ICEs (Garriss et al., 2009, 2013). Similarly, IncA/C plasmids could enhance their plasticity by recombining with a replicon present in the same cell that shares short identical sequences. Altogether, these observations give glimpses of the high dynamics of IncA/C plasmids and their impact on genome plasticity, which could have significant implications for pathogenic bacteria and forecast a bleak future for antibiotherapies.

Many questions remain regarding the coexistence in *V. cholerae* of IncA/C plasmids and SXT/R391 ICEs as two different yet related entities capable of conferring MDR and in particular, resistance to co-trimoxazole. For instance, no single isolate bearing both types of element has been described to date with the exception of isolates from Eastern China that were found to bear both pMRV150-like IncA/C plasmids and SXT-like elements (Pan et al., 2008). Although the IncA/C plasmids in these strains were shown to be able to transfer to recipient cells, no exconjugant bearing also a copy of the SXT-like elements was found, thereby suggesting that the latter were not functional or that negative interference between the two families of mobile elements occurs. Given these observations, we are wondering whether there is a mutual exclusion of IncA/C plasmids and SXT/R391 ICEs. If so, given the relative efficiency of transfer of both types of mobile genetic elements, can we expect to observe a displacement of SXT/R391 ICEs by IncA/C plasmids in clinical and environmental populations of *V. cholerae* in the near future? Given the prevalence of IncA/C plasmids in a plethora of bacteria, their broad host-range and their ability of mobilize MDR-conferring genomic islands, close attention needs to be paid concerning their circulation and evolution. Sequencing of pathogenic isolates bearing IncA/C plasmids and sequences analyses provide valuable information regarding the epidemiology of IncA/C plasmids but molecular characterization of their mechanism of transfer remain unavoidable to unravel the characteristics that make them so successful in modern pathogens. Using pVCR94Δ X as a prototype, it is now easier to explore the biology and regulation of IncA/C plasmids.

### Conflict of interest statement

The authors declare that the research was conducted in the absence of any commercial or financial relationships that could be construed as a potential conflict of interest.
